# Whole blood resuscitation and post-traumatic organ dysfunction in injured children

**DOI:** 10.1111/trf.70169

**Published:** 2026-03-12

**Authors:** Erin V. Feeney, Madelyn A. Scarmack, Leah M. Furman, Philip C. Spinella, Anne H. Kalinowski, Christopher M. Horvat, Barbara A. Gaines, Christine M. Leeper

**Affiliations:** 1 Department of Surgery, University of Pittsburgh, Pittsburgh, Pennsylvania, USA; 2 School of Medicine, Lake Erie College of Medicine, Erie, Pennsylvania, USA; 3 Department of Critical Care, Children’s Hospital of Pittsburgh, Pittsburgh, Pennsylvania, USA; 4 Department of Surgery, University of Texas Southwestern, Dallas, Texas, USA

**Keywords:** hemostatic resuscitation, organ dysfunction, pediatric trauma, whole blood

## Abstract

**Background:**

Both whole blood (WB) and component therapy (CT) are used for hemostatic resuscitation in injured children. We hypothesize that early WB transfusion compared to CT alone is associated with decreased post-traumatic organ dysfunction.

**Study Design and Methods:**

This single-center observational study included children ages 0–17 years between January 2021 and March 2024 with trauma mechanism and intensive care unit admission. The primary outcome was Pediatric Logistic Organ Dysfunction 2 (PELOD-2) score on post-trauma days 1–7. Data were analyzed using linear regression adjusting for age, sex, race, year, injury mechanism, injury severity score (ISS), shock index pediatric age-adjusted, and total 4-h transfusion volume (mL/kg).

**Results:**

In total, 540 subjects met eligibility criteria; of the 52/540 (10%) who received blood transfusion within 4 h, 11/52 (21%) received RBC alone, 12/52 (23%) received WB alone, 9/52 (17%) of subjects received RBC plus other, and 20/52 (38%) received WB plus other. The cohort was 60% (326/540) male, 83% (449/540) blunt injury mechanism, median (interquartile range [IQR]) age 3 years (0–11), and median (IQR) ISS of 11 (8–18). In adjusted analysis, transfusion of WB + other was an independent predictor of lower PELOD-2 score through post-trauma day 7 in comparison to subjects receiving RBC + other.

**Discussion:**

In subjects who were transfused multiple blood products, receipt of any WB versus CT alone for hemostatic resuscitation after injury was associated with reduced organ dysfunction. Further investigation is needed in large cohorts to fully elucidate clinical benefit and improve mechanistic understanding.

## 1 | INTRODUCTION

Organ dysfunction following post-traumatic hemorrhage is a significant contributor to morbidity and mortality in pediatric trauma, the leading cause of death in children and young adults.^1–6^ Despite a physiologic response to trauma that is distinct in children, hemostatic resuscitation guidelines are often derived from adult data due to a paucity of high-quality pediatric-specific data.^1,2^ Current consensus guidelines recommend resuscitation with either component therapy (CT) or low titer O whole blood (WB) in the setting of life-threatening hemorrhage.^3^ While WB is less commonly available at pediatric trauma centers compared to adult centers, its use in pediatric trauma is increasing.^4^ Investigation into the optimal approach to hemostatic resuscitation, generated from pediatric-specific data, is critical in order to reduce the burden of preventable morbidity and mortality in injured children.

Post-traumatic organ dysfunction, a commonly seen clinical presentation, is associated with worse functional outcomes and higher rates of mortality.^2,5–7^ In large registry studies, 49% of pediatric subjects admitted to the intensive care unit (ICU) had one or more organ systems with organ dysfunction, and 23% of subjects met criteria for multiple organ dysfunction syndrome.^7^ The mortality rate for subjects with multiple organ dysfunction was 20.1% compared to 10.3% in those without.^7^ Furthermore, organ dysfunction is a particularly relevant outcome in patients who present with life-threatening hemorrhage and subsequent coagulopathy; subjects who presented with trauma-induced coagulopathy developed organ dysfunction at a rate of 68.4% versus 7.7% in those without coagulopathy (*p* < 0.01).^5^

There is a growing body of literature to support the safety and potential benefits of WB in children.^8–20^ However, the impact of blood product type (WB vs. CT) on the development of post-traumatic organ dysfunction following emergent transfusion is not well elucidated in pediatric cohorts. We hypothesize that, in a cohort of injured children admitted to the ICU after receiving emergent blood transfusion, resuscitation with WB will be associated with decreased incidence of post-traumatic organ dysfunction compared to CT.

## 2 | METHODS

### 2.1 | Data source and study population

This is an observational study of injured children ages 0–17 years presenting to a single trauma center between January 2021 and March 2024. The institutional trauma registry was queried for all subjects meeting the following inclusion criteria: traumatic injury mechanism and admission to the ICU. ICU admission was selected to include a cohort with high injury severity and to exclude children who did not survive their initial trauma bay resuscitation in order to reduce survival bias. Subjects who did not receive early transfusion (within 4 h of arrival) but did receive either WB or RBCs at greater than 4 h following arrival were excluded.

### 2.2 | Outcomes and definitions

The weight-based volume for all blood products transfused within 4 h (WB, RBC, plasma, platelets, and cryoprecipitate) was recorded for all subjects. Subjects were categorized based on transfusion strategy: no transfusion, RBC alone, WB alone, RBC plus other, or WB plus other. The primary outcome was Pediatric Logistic Organ Dysfunction 2 (PELOD-2) score^21^ on post-trauma days 1–7. Daily PELOD-2 score was calculated in an automated fashion from retrospective review of a structured ICU data pull. If multiple datapoints used for score calculation were recorded for the same day, the value with furthest deviation from normal was included. Subjects with mortality or discharge prior to post-trauma day 7 were assigned the minimum PELOD-2 score (0) following discharge and the maximum PELOD-2 score (33) following mortality. Missing values were presumed to be within normal range.

### 2.3 | Statistical analysis

Descriptive statistics were calculated as number of patients (%) for categorical variables and median (interquartile range [IQR]) for continuous variables. Univariate comparisons were made using Mann–Whitney and Kruskal–Wallis tests for comparisons as appropriate. The association between blood product transfusion strategy and PELOD-2 score was analyzed for post-trauma days 1–7 using multivariable linear regression adjusting for the following a priori selected variables: age, sex, race, injury mechanism, year, injury severity score (ISS), shock index pediatric age-adjusted (SIPA), and total 4-h transfusion volume (mL/kg). Subjects meeting eligibility criteria who did not receive transfusion served as the reference group. Variation inflation factors were calculated to assess for collinearity and adjusted *R*^2^ values were used to assess model fit. Coefficients and 95% confidence interval were reported. Statistical analysis was performed with STATAv18 (Stata Corp, College Station, TX). Statistical significance was defined as a two-tailed *p*-value of <0.05.

### 2.4 | Ethics and regulatory

This study was conducted under expedited approval from the Institutional Review Board (STUDY22070093). This manuscript has been prepared in accordance with the Strengthening the Reporting of Observational studies in Epidemiology (STROBE) guidelines.

## 3 | RESULTS

**FIGURE 1:**
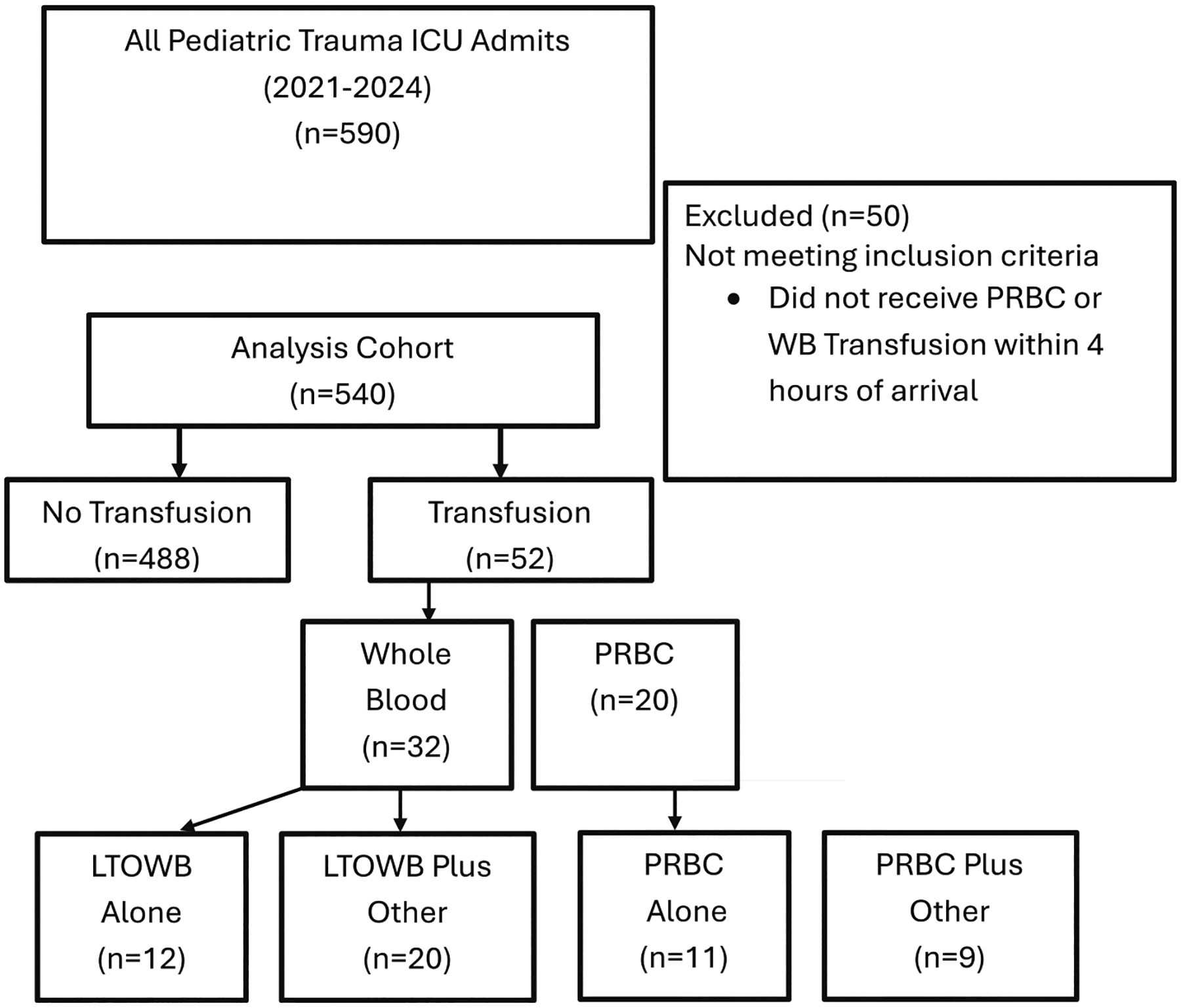
Analysis cohort selection.

In total, 540 subjects met eligibility criteria of whom 52/540 (10%) received blood product transfusion within 4 h (Figure 1). Of those subjects receiving blood product transfusion, 11/52 (21%) received RBC alone, 12/52 (23%) received WB alone, 9/52 (17%) of subjects received RBC plus other, and 20/52 (38%) received WB plus other. The cohort was 326/540 (60%) male, 449/540 (83%) blunt traumatic injury mechanism, with median (IQR) age of 3 years (0–11) and median (IQR) ISS of 11 (8–18). In comparison to subjects receiving either RBC or WB in isolation, subjects receiving RBC plus other or WB plus other had a higher median (IQR) ISS (30 (26–38) versus 22 (14–27)) (Table 1).

On unadjusted analysis, PELOD-2 scores were significantly different between transfusion strategy cohorts on post-trauma days 1–7, with those receiving either WB plus other or RBC plus other consistently demonstrating higher scores than subjects receiving WB or RBCs in isolation (Table 2). In an adjusted multivariate linear regression model, transfusion strategy was an independent predictor of PELOD-2 score from post-trauma day 1 through post-trauma day 7; PELOD-2 scores for recipients of WB plus other were significantly lower than PELOD-2 scores for recipients of RBC plus other (Table 3). No statistically significant association to post-trauma PELOD-2 scores was observed in subjects who received RBCs or WB in isolation.

## 4 | DISCUSSION

In this single-site analysis assessing the impact of hemostatic resuscitation transfusion strategy on the development of post-traumatic organ dysfunction in children admitted to the ICU, receipt of WB as part of early transfusion strategy compared to CT alone was associated with reduced end-organ dysfunction for children receiving multiple blood products. These data further support the potential clinical benefits for the use of WB compared to CT in severely injured children. Although these findings do not elucidate the mechanism by which WB confers benefit, they support the principle that a resuscitation strategy impacting end-organ perfusion and correction of coagulopathy can have a measurable clinical effect on post-traumatic organ dysfunction.

These data support the concept that all patients likely do not benefit equally from WB resuscitation. In studies of children receiving massive transfusion, WB was reported to confer a survival advantage^22^; this benefit is inconsistent in studies that include all recipients of WB, particularly when administered in lesser quantities.^13,18,23^ Subjects receiving WB or RBCs in isolation represent a unique subset of patients who meet criteria for transfusion on arrival but do not present with the same degree of clinically significant hemorrhage as subjects who require additional blood product components. It is possible that for children who require a single dose or unit of blood product, the type of resuscitation selected is less impactful than for those who are severely injured, in shock, and require multiple blood products. This analysis suggests that for children who receive massive transfusion, that is, an emergency release RBC or WB unit and then require additional product, the use of WB may confer an advantage regarding later development of organ dysfunction. While the precise mechanism cannot be determined from this dataset, it is possible that the efficiency in administration and rapid mitigation of shock and coagulopathy underly this benefit.^13^

Existing data regarding the impact of resuscitation strategy on organ dysfunction in pediatric trauma cohorts are limited. One single-site retrospective study examined PELOD-2 score as a secondary outcome in their assessment of the safety of WB use in pediatric trauma cohorts. This study reported that in a cohort of 36 pediatric trauma subjects receiving WB in addition to CT, propensity match analysis to a historical control cohort of subjects receiving conventional CT, no difference in PELOD-2 scores on post-trauma days 3 and 7 was observed.^23^ This study did not differentiate by volume or category of products received (e.g., RBC alone versus RBC plus other products) and was limited by small cohort size and low incidence of post-traumatic organ dysfunction. Another study utilizing the American College of Surgeons—Trauma Quality Improvement Program database developed a composite outcome for organ dysfunction that included mechanical ventilation greater than 6 days, acute respiratory distress syndrome (ARDS), acute kidney injury, myocardial infarction, or nosocomial infection (sepsis, catheter-associated urinary tract infection or ventilator associated pneumonia). In this cohort of 6237 subjects receiving either WB or CT within 4 h of arrival to emergency department, receipt of any WB transfusion compared to CT alone was associated with decreased odds of organ dysfunction (odds ratio 0.78 (95% CI 0.61–0.98; *p* = 0.04)) after adjusting for age, sex, trauma mechanism, ISS, head abbreviated injury score, Glasgow Coma Scale (GCS), shock, interfacility transfer, 4-h weight-adjusted volume of blood products. While this analysis utilized a different primary outcome for organ dysfunction due to the lack of granularity in the database, the clinical effect of WB in decreasing post-traumatic organ dysfunction is congruent with our findings.

In adult trauma populations, studies evaluating the relationship between transfusion strategy and subsequent organ dysfunction are also limited and have produced mixed findings. Analyses of large national trauma registries have suggested an association between WB transfusion and increased rates of severe sepsis (defined as sepsis with associated end-organ dysfunction) and ARDS.^24,25^ Limitations of these studies include the potential influence of survival bias, the assumption that missing data were missing completely at random, and a lack of granularity to quantify the severity and timing of organ dysfunction. However, these findings highlight the possibility that the physiologic effects of WB transfusion on organ dysfunction may differ in pediatric versus adult populations and underscore the need for ongoing rigorous investigation in both adult and pediatric trauma cohorts.

## 5 | LIMITATIONS

This study is limited by its single-site and retrospective design. While we sought to control for the effect of confounding in our model, the small analysis cohort size along with the relative infrequency of the intervention of interest limited our ability to account for all bias and prohibited robust subgroup analysis. The study population presented in this analysis includes pediatric trauma patients admitted to the ICU and is therefore not generalizable to all injured cohorts. Notably, subjects who did not survive to admission to the ICU, including death in the trauma bay, are not captured in this analysis, limiting generalizability of this data. The use of PELOD-2 as an outcome for organ dysfunction also has some inherent limitations. This scoring system was derived from data that included subjects admitted to the PICU with critical illness of any etiology (medical, surgical, or trauma),^21^ and while the PELOD-2 score has been validated in pediatric sepsis literature,^26–28^ its ability to predict mortality has not been broadly validated in pediatric trauma cohorts.^29^ We have selected PELOD-2 score through day 7 as an outcome to quantify organ dysfunction, which may not capture organ dysfunction that occurs later in the hospital course.

## 6 | CONCLUSION

Receipt of WB as part of early hemostatic resuscitation was independently associated with lower post-traumatic organ dysfunction scores in a subset of critically injured children who required multiple blood product transfusions. Further investigation is needed in large, prospective cohorts to fully elucidate clinical benefit and improve mechanistic understanding.

## Supplementary Material

Supplementary table 1

STROBE checklist

Additional supporting information can be found online in the Supporting Information section at the end of this article.

## Figures and Tables

**TABLE 1:** Cohort characteristics and clinical outcomes.

	Total (*N* = 540)	No transfusion (*N* = 488)	RBC only (*N* = 11)	WB only (*N* = 12)	RBC + other (*N* = 9)	WB + other (*N* = 20)	*p*-value
Subject characteristics							
Age, median (IQR)	3 (0–11)	2 (0–11)	3 (0–9)	12 (4–16)	9 (3–13)	6 (2–13)	0.01
Sex, frequency (%)							
Male	326 (60)	294 (60)	6 (55)	8 (67)	5 (56)	13 (65)	0.96
Female	214 (40)	194 (40)	5 (45)	4 (33)	4 (44)	7 (35)	
Race, frequency (%)							
Asian	2 (0.4)	2 (0.4)	0 (0)	0 (0)	0 (0)	0 (0)	0.62
Black	107 (20)	98 (20)	0 (0)	1 (8)	3 (33)	5 (25)	
White	378 (70)	338 (69)	10 (91)	10 (83)	5 (56)	15 (75)	
Other^a^	9 (2)	8 (2)	0 (0)	0 (0)	1 (11)	0 (0)	
Unknown	44 (8)	42 (9)	1 (9)	1 (8)	0 (0)	0 (0)	
Mechanism of injury							
Blunt	483 (89)	413 (85)	9 (82)	10 (83)	7 (78)	17 (85)	0.02
Anoxic (drowning/hanging)	26 (5)	25 (5)	0 (0)	0 (0)	1 (11)	0 (0)	
Penetrating	19 (4)	12 (2)	1 (9)	2 (17)	1 (11)	3 (15)	
Burns	12 (2)	11 (2)	1 (9)	0 (0)	0 (0)	0 (0)	
Injury severity score, median (IQR)	11 (8–18)	10 (8–17)	25 (13–29)	21 (16–27)	27 (26–34)	32 (25–38)	<0.01
Blood product transfusion, median (IQR)							
4-h whole blood transfusion volume (mL/kg)	—	—	—	10 (7–16)	—	20 (10–34)	<0.01
4-h total transfusion volume (mL/kg)	—	—	7 (3–12)	10 (7–16)	35 (33–36)	44 (20–79)	<0.01
4-h whole blood: TTV ratio	—	—	—	—	—	0.5 (0.3–0.6)	<0.01
24-h total transfusion volume (mL/kg)	—	—	8 (6–20)	13 (8–17)	49 (44–60)	62 (24–110)	
Clinical outcomes, frequency (%)							
24-h mortality	8 (1)	4 (0.01)	0 (0)	0 (0)	1 (11)	3 (15)	<0.01
7-day mortality	20 (4)	6 (0.01)	0 (0)	0 (0)	5 (56)	9 (45)	<0.01
In-hospital mortality	24 (4)	9 (2)	0 (0)	0 (0)	5 (56)	10 (50)	<0.01
7-day discharge (alive)	440 (81)	425 (87)	5 (45)	9 (75)	0 (0)	1 (5)	<0.01
Hospital length of stay, days	2 (1–5)	2 (1–4)	9 (5–14)	5 (2–10)	4 (2–19)	7 (3–17)	<0.01
Ventilator days	0 (0–0)	0 (0–0)	0 (0–0)	0 (0–0)	0 (0–0)	0 (0–1)	<0.01

a Other denotes transfusion of any plasma, platelets, and cryoprecipitate in addition to either WB or RBC.

**TABLE 2:** Unadjusted pediatric logistic (PELOD-2) scores by transfusion strategy category on post-trauma days 1 through 7.

	Total (*N* = 540)	No transfusion (*N* = 488)	RBC only (*N* = 11)	WB only (*N* = 12)	RBC + other (*N* = 9)	WB + other (*N* = 20)
PELOD-2^a^ score						
Day 1 (*n* = 540)	2 (0–4)	2 (0–3)	6 (1–7)	3 (0–5)	15 (11–18)	11 (8–17)
Day 2 (*n* = 520)	2 (0–5)	2 (0–3)	7 (2–10)	2 (0–5)	13 (8–19)	9 (6–17)
Day 3 (*n* = 382)	0 (0–4)	0 (0–2)	3 (0–9)	1 (0–6)	10 (8–15)	9 (6–14)
Day 4 (*n* = 251)	0 (0–5)	0 (0–3)	0 (0–6)	0 (0–4)	9 (7–22)	10 (6–15)
Day 5 (*n* = 177)	0 (0–5)	0 (0–3)	0 (0–4)	0 (0–9)	9 (7–12)	8 (0–13)
Day 6 (*n* = 142)	0 (0–4)	0 (0–3)	0 (0–4)	0 (0–6)	8 (4–11)	9 (3–10)
Day 7 (*n* = 114)	0 (0–4)	0 (0–3)	0 (0–4)	0 (0–0)	6 (2–11)	9 (3–12)

a Pediatric Logistic Organ Dysfunction 2 score. Reported as median (interquartile range [IQR]).

**TABLE 3:** Multivariate linear regression model PELOD-2 post-trauma days 1–7.

*N* = 540	Contrast^a^	95% conf. interval	*p*-value
Post-trauma day 1^b^			
RBC + other versus WB + other	3.61	0.69–6.53	0.02
Post-trauma day 2			
RBC + other versus WB + other	4.92	1.50–8.35	<0.01
Post-trauma day 3			
RBC + other versus WB + other	5.00	1.53–8.47	<0.01
Post-trauma day 4			
RBC + other versus WB + other	8.21	4.65–11.78	<0.01
Post-trauma day 5			
RBC + other versus WB + other	7.88	3.91–11.84	<0.01
Post-trauma day 6			
RBC + other versus WB + other	9.04	5.10–12.98	<0.01
Post-trauma day 7			
RBC + other versus WB + other	8.44	4.41–12.46	<0.01

a Differences between adjusted predicted means were estimated using margins with pairwise contrasts.

b Variables adjusted for in this model include sex, age, injury mechanism, injury severity score, shock index pediatric age-adjusted (SIPA), 4-h total transfusion volume, race, and year.

## Data Availability

Data use agreement prohibits sharing of data.

## Abbreviations:

ARDS: acute respiratory distress syndrome
CT: component therapy
GCS: Glasgow Coma Scale
ICU: intensive care unit
ISS: injury severity score
PELOD-2: Pediatric Logistic Organ Dysfunction 2
SIPA: shock index, pediatric age-adjusted
WB: low titer O whole blood
