# Supplementary material for: Whole blood resuscitation and post‐traumatic organ dysfunction in injured children
Source: Transfusion. 2026 Mar 12;66(Suppl 1):S66–73. doi: 10.1111/trf.70169 (PMC13102025; doi:10.1111/trf.70169)
Supplement: Supplementary file 1 — Table S1. Multivariate linear regression model PELOD‐2 post‐trauma days 1–7. [file TRF-66-S66-s001.docx]

| N=540 | Coefficient | 95% Conf. Interval | p-value | |  |  |
| --- | --- | --- | --- | --- | --- | --- |
| Post-Trauma Day 1 |  |  | |  | |  |
| RBC Plus Other | 7.51 | 4.88-10.15 <0.01 | |  | |  |
| WB Plus Other | 3.90 | 1.37-6.44 <0.01 | |  | |  |
| RBC Alone | 0.90 | -1.48-3.29 0.46 | |  | |  |
| WB Alone | -0.11 | -2.21-1.98 0.92 | |  | |  |
| Post-Trauma Day 2 |  |  | |  | |  |
| RBC Plus Other | 9.61 | 6.52-12.71 <0.01 | |  | |  |
| WB Plus Other | 4.69 | 1.71-7.67 <0.01 | |  | |  |
| RBC Alone | 1.23 | -1.57-4.03 0.39 | |  | |  |
| WB Alone | -0.37 | -2.83-2.08 0.77 | |  | |  |
| Post-Trauma Day 3 |  |  | |  | |  |
| RBC Plus Other | 11.66 | 8.52-14.79 <0.01 | |  | |  |
| WB Plus Other | 6.65 | 3.63-9.67 <0.01 | |  | |  |
| RBC Alone | 1.43 | -1.40-4.27 0.32 | |  | |  |
| WB Alone | 0.12 | -2.37-2.61 0.92 | |  | |  |
| Post-Trauma Day 4 |  |  | |  | |  |
| RBC Plus Other | 14.43 | 11.21-17.65 <0.01 | |  | |  |
| WB Plus Other | 6.22 | 3.12-9.32 <0.01 | |  | |  |
| RBC Alone | 0.46 | -2.45-3.37 0.76 | |  | |  |
| WB Alone | -0.70 | -3.25-1.86 0.59 | |  | |  |
| Post-Trauma Day 5 |  |  | |  | |  |
| RBC Plus Other | 15.89 | 12.30-19.47 <0.01 | |  | |  |
| WB Plus Other | 8.01 | 4.56-11.45 <0.01 | |  | |  |
| RBC Alone | -0.70 | -3.93-2.54 0.67 | |  | |  |
| WB Alone | -0.45 | -3.29-2.40 0.76 | |  | |  |
| Post-Trauma Day 6 |  |  | |  | |  |
| RBC Plus Other | 14.38 | 10.82-17.94 <0.01 | |  | |  |
| WB Plus Other | 5.34 | 1.92-8.76 <0.01 | |  | |  |
| RBC Alone | -0.89 | -4.11-2.32 0.59 | |  | |  |
| WB Alone | -1.47 | -4.30-1.35 0.31 | |  | |  |
| Post-Trauma Day 7 | |  |  | |  | |
| RBC Plus Other | | 13.98 | 10.35-17.62 <0.01 | |  | |
| WB Plus Other | | 5.55 | 2.05-9.05 <0.01 | |  | |
| RBC Alone | | -0.78 | -4.06-2.51 0.64 | |  | |
| WB Alone | | -1.95 | -4.84-0.93 0.18 | |  | |
| *Variables adjusted for in this model include sex, age, injury mechanism, injury severity score, shock index pediatric age-adjusted (SIPA), 4-hour total transfusion volume, race, and year. Subjects who were not transfused serve as the reference group. | | | |  | |  |

**Supplemental Table 1** **Multivariate Linear Regression Model PELOD-2 Post-Trauma Days 1-7**
